# Brain Disorder Detection and Diagnosis using Machine Learning and Deep Learning – A Bibliometric Analysis

**DOI:** 10.2174/1570159X22999240531160344

**Published:** 2024-05-31

**Authors:** Jyotismita Chaki, Gopikrishna Deshpande

**Affiliations:** 1 School of Computer Science and Engineering, Vellore Institute of Technology, Vellore, India;; 2 Department of Electrical and Computer Engineering, AU MRI Research Center, Auburn University, AL, USA;; 3 Department of Psychological Sciences, Auburn University, AL, USA;; 4 Alabama Advanced Imaging Consortium, Birmingham, AL, USA;; 5 Center for Neuroscience, Auburn University, AL, USA;; 6 School of Psychology, Capital Normal University, Beijing, China;; 7 Key Laboratory for Learning and Cognition, Capital Normal University, Beijing, China;; 8 Department of Psychiatry, National Institute of Mental Health and Neurosciences, Bangalore, India;; 9 Centre for Brain Research, Indian Institute of Science, Bangalore, India;; 10 Department of Heritage Science and Technology, Indian Institute of Technology, Hyderabad, India

**Keywords:** Brain disorder, machine learning, deep learning, Alzheimer’s, Parkinson’s, autism

## Abstract

**Background and Objective:**

Brain disorders are one of the major global mortality issues, and their early detection is crucial for healing. Machine learning, specifically deep learning, is a technology that is increasingly being used to detect and diagnose brain disorders. Our objective is to provide a quantitative bibliometric analysis of the field to inform researchers about trends that can inform their Research directions in the future.

**Methods:**

We carried out a bibliometric analysis to create an overview of brain disorder detection and diagnosis using machine learning and deep learning. Our bibliometric analysis includes 1550 articles gathered from the Scopus database on automated brain disorder detection and diagnosis using machine learning and deep learning published from 2015 to May 2023. A thorough bibliometric análisis is carried out with the help of Biblioshiny and the VOSviewer platform. Citation analysis and various measures of collaboration are analyzed in the study.

**Results:**

According to a study, maximum research is reported in 2022, with a consistent rise from preceding years. The majority of the authors referenced have concentrated on multiclass classification and innovative convolutional neural network models that are effective in this field. A keyword analysis revealed that among the several brain disorder types, Alzheimer's, autism, and Parkinson's disease had received the greatest attention. In terms of both authors and institutes, the USA, China, and India are among the most collaborating countries. We built a future research agenda based on our findings to help progress research on machine learning and deep learning for brain disorder detection and diagnosis.

**Conclusion:**

In summary, our quantitative bibliometric analysis provides useful insights about trends in the field and points them to potential directions in applying machine learning and deep learning for brain disorder detection and diagnosis.

## 1 INTRODUCTION

The evolution and development of healthcare systems have become an important aspect of the medical field. Detecting diseases has also grown more reliant on biomedical technology such as ultrasonography, X-rays, particle beams, and MRI, among others [1]. The excessive accumulation of biological data is a challenge for healthcare providers as technology is used more. Nonetheless, high-performance computer technologies have accelerated the analysis of biological data and lowered the workload on healthcare workers. Adoption of technology has helped us understand various human diseases including cardiovascular, genetic, psychological, brain, skin, trauma, infectious, tissue, and digestive issues, to name a few [2].

Despite advances in medical technology and care, diagnoses of brain disorders (especially psychiatric disorders) remain a challenge due to our limited understanding of brain function. The brain is often regarded as the most essential organ in the human body, governing ideas, memories, emotions, motor abilities, vision, and respiration. The human brain is made up of 100 billion neurons that are linked together by more than 100 trillion synapses [3]. These connections are organized at different spatial scales anatomically, and interact with one another at different temporal scales functionally. Because of this complexity, understanding the neurological underpinnings of brain functions remains a challenge.

Since the brain is such an essential organ, brain disorders have a large burden on one’s life. People's memory, senses, and even the personality can all be negatively impacted by the brain disorder [4]. Although increased awareness of these diseases and technological/scientific progress has reduced mortality, some chronic brain disorders can cause permanent or partial impairment or pain. The global prevalence of these diseases was 15% of all cases [5]. Furthermore, these disorders have a high annual causation rate of 16.8%.

Brain disorder diagnosis is a growing and challenging issue due to the large volumes of brain disorder data generated by current diagnostic tools. Manual analysis of this data is often subjective, leading to the development of automated computer-aided diagnosis systems using machine learning (ML) and deep learning (DL) technology [6-8]. These systems have become an important research topic in recent years, as medical data collection techniques and ML approaches may vary depending on the diagnostic method and disorder type. The field has seen explosive growth in recent years, making it difficult to understand the path charted due to the large and diverse literature. A systematic bibliometric study of the field is needed to assess trends, future research subjects, state-of-the-art, and breakthroughs in brain disorder detection and diagnosis using ML and DL techniques.

The motivation of this study is to enhance past evaluations with a bibliometric review of ML and DL-based brain disorder detection and diagnosis. Bibliometric research is a quantitative and statistical examination of literature that allows for the investigation of far bigger bibliographic datasets than qualitative systematic literature studies. Bibliometric studies have grown in popularity in recent years as a result of their benefits. In recent years, bibliometric methodologies have been employed in a wide range of fields and subjects [9-11] assist in to progress of a topic by gathering and analyzing earlier research and methodically summariz- ing existing results and can also assist in defining possible future research topics, providing a forum for interested re- searchers. The second objective of this research is to identify the most significant entities in this sector, such as influential countries, institutions, sources, and publications as well as to identify research gaps in the field of ML and DL-based brain disorder detection and diagnosis which can lead to indicate future research priorities.

The purpose of this bibliometric research is to address the following questions to expand earlier research on ML and DL in brain disorder detection, and diagnosis:

1. What are the most reputable sources, materials, and most often referenced papers in this field?
2. How many average and year-by-year citations for each research document receive?
3. What is the yearly research growth rate and the link between countries, authors, and research papers?
4. Which journals are the most successful and frequently cited as well as which countries have made the biggest contributions in the aforementioned field?
5. Who are the field's most influential and most often mentioned authors and which are the most influential institutions?
6. What is the link between the number of papers published and the number of authors in the most trending topic in this field?
7. Which keywords are often used?
8. What are some prospective future research directions that might aid in the advancement of research on ML and DL-based brain disorder detection and diagnosis?

The above research questions reveal changes in research output over time, influencing funding decisions and future research orientations. By analyzing citation effects of publications, and journals, scholars can identify significant research on a subject [12-14]. Understanding collaboration patterns can the researchers identify successful research partnerships, reveal new research areas, and provide insights into productivity and impact across different areas and nations, driving research policy and funding decisions.

This research contributes to the field of ML and DL-based brain disorder detection and diagnosis by identifying research trends, creating a bibliometric framework for analysis, and identifying networks of collaboration among authors, institutions, and countries [15-17]. It helps identify gaps in the literature and guides future research strategies. Practical contributions include identifying essential study areas for other researchers to prioritize and identifying important researchers and institutions for collaboration. This data can help researchers identify gaps in the literature and encourage knowledge sharing among researchers.

In our investigation, we combined two tools: Bibliometrix/Bilioshiny [18] and VOSviewer [19]. To begin, Bibliometrix is a free and open-source R program created by Massimo Aria and Corrado Cuccurullo. It enables a wide range of different types of analysis on bibliometric data. Bibliometrics was also supplemented by Biblioshiny. Biblioshiny improves the generation of bibliometric data visualizations. VOSviewer was used alongside Biblioshiny and Bibliometrix. VOSviewer is a bibliometric data visualization tool developed by the Centre for Science and Technology Studies at Leiden University in the Netherlands. VOSviewer has been used in several bibliometric studies and allows for the creation of bibliometric networks that demonstrate associations between, for example, publications, keywords, or researchers. VOSviewer also allows for the establishment of co-citation, bibliographic coupling, and co-authorship analysis. Although Biblioshiny excels in statistical features, we found VOSviewer to be an excellent tool for visualizing keyword co-occurrences.

The organization of the manuscript is as follows: section 2 provides the overview of materials and methods, results of the analysis are reported in section 3, the discussion and the details of the future research agenda related to the analysis are reported in section 4, and at last the conclusion section concludes the manuscript.

## 2 MATERIALS AND METHODS

This section describes our bibliometric methodology [20-22]. The process of conducting bibliometric research may be separated into three parts. First, the data to be analyzed must be gathered. The first subsection describes this phase. The data-collecting process is followed by the data analysis step. The second subsection describes this procedure. The third subsection describes the bibliometric analysis methodology.

### 2.1 Bibliometric Data Collection

The initial step was to gather bibliometric information for our investigation. Several databases exist now for the collection of bibliometric data, with Scopus and Web of Science being among the most popular. The characteristics and functionality of these databases differ and thus we collected the bibliometric data from only the Scopus [23] database. The main motivation for selecting the Scopus database is as follows. This database includes more journals than Web of Science and was thus deemed to be appropriate for identifying as many research articles as feasible. Although additional databases such as Google Scholar and PubMed are available, we choose not to use them [24]. First, unlike Google Scholar, Scopus allows researchers to create a comprehensive search phrase and instantly obtain all bibliometric metadata. Second, Scopus covers far more multidisciplinary research than PubMed. Thus, we identified Scopus to be the best database for conducting a bibliometric study since ML and DL-based brain disorder detection and diagnosis is a multidisciplinary research field.

We conducted a subject data search that comprised the title, abstract, and keywords on 1^st^ June 2023. We discovered that many titles did not fit our desired keywords even when the corresponding papers investigated topics relevant to the scope of our search. For example, instead of referring to “deep learning” and “machine learning,” authors would refer to a specific deep learning technique, such as the convolutional neural network (CNN). Some researchers didn't even include “brain disorder” in the title, rather they have used the name of the disorder such as Alzheimer’s, Parkinson's, *etc*. This led to the following search string that was applied: ((“machine learn*” OR “deep learn*” OR “neural network”) AND (“brain disord*”) AND (“detection” OR “diagnosis” OR “classification”). The search results were narrowed down to 8.5 years (2015-May 2023) to find the most recent trends and state of the art. The justification for taking into consideration the articles from 2015 is as follows: before 2015 there was no significant number of articles published in the considered domain. Because of Scopus' syntax, the * sign is used to search for all potential word ends of the search query. An additional filter was used, which restricted the document type to the article, conference paper, review, book chapter, and book. Finally, a total of 1550 Scopus document entries with all associated metadata were exported. Fig. (**1**) shows the pictorial depiction of the bibliometric data collection method.

### 2.2 Data Analysis

Bibliometric analysis techniques are emerging techniques nowadays as it provides the trend and importance of a particular research field. In thus study two tools Bibliometrix/Bilioshiny [18] and VOSviewer [19] are used for the analysis as discussed in the introduction section.

### 2.3 Bibliometric Analysis Methodology

Bibliometric analysis is an academic literature evaluation approach that depends on the quantitative examination of publications.

We begin by providing a summary of the dataset, which includes the following items:

- Main information and general matrices: A table displaying the key characteristics of our dataset.
- Scientific production: a diagram representing the investigated field's yearly scientific productivity.
- Average citations per year: An illustration depicting the increase of average citations over time.
- Three-field plot: Relationship between authors, countries, and titles.

The second section of the findings is regarding sources. This is based on a domain analysis and visualization approach. The source in bibliometric analysis typically includes several components These components can call attention to potentially relevant trends and patterns, as well as scientific transformation concepts that can influence conceptual frameworks. We concentrated on the following concerns in this step:

- The most productive and top cited sources: A visualization displaying the top ten most active journals in terms of the number of papers published and the top cited journals in terms of the number of citations received.
- Sources based on Bradford’s law: A pictorial depiction of sources which is based on Bradford’s law [25].
- Sources production over time: A graphic that represents the sources' publication amount over time.

The third part is related to the detail visualization of the information related to the authors. In this section we have covered the following:

- The Most Relevant Authors: a graphic displaying the top ten writers in terms of article volume.
- The Most Relevant Institutions: A graph depicting the top ten most productive organizations based on the number of papers generated.
- The most relevant countries: A diagram displaying the top ten most relevant nations based on the number of articles generated, either in single-country or multiple-country publications.
- The Most Cited Authors: A diagram of the top ten most cited authors from the Scopus database.
- Author productivity through Lotka's Law: A diagram and table displaying the frequency analysis of research publications based on Lotka's law [26].
- Most cited countries: A graph depicting the most cited countries.

The fourth subsection is based on the detail representation related to documents. In this part we have covered the following:

- The Most Cited documents: A graph of the top ten most cited publications from the Scopus database.
- The Most Frequent Words: A visualization and a word cloud of the most frequently used words in the documents' abstract, title, and keywords.
- Word’s frequency over time: A graph that represents the most frequently used words over time.
- Trend topics: A representation of the hot topics in the field.
- Most local cited references: A visualization of the most cited references collected from the selected publications.

The fifth part presents details of the conceptual structure. In this part we have focused on the following:

- Keyword co-occurrence network: A graph that shows the association between important terms and separates them into smaller groupings.
- Thematic Map: A thematic map is a network graph that is generated by the keywords and how they are related. The labels of each thematic map are recognized by the name of the keyword that appears most frequently in the associated topic [27].
- Factorial analysis: The factorial analysis is done based on Correspondence Analysis (CA) and Multiple Correspondence Analysis (MCA). CA [28] is a visual approach to understanding the relationship between elements in a frequency table is correspondence analysis. It is a derivative of principal component analysis and is meant to analyze links between qualitative factors. MCA [29] is used for analyzing the data visually, multidimensionally, and mathematically.

The final section is network analysis. Network analysis, according to graph theory or network theory, is the study of network characteristics and the interactions between their vertices and arcs. The betweenness centrality indices and proximity are the most important measurements for network analysis [30]. We concentrated on the following items in this section of the research:

- Collaboration globe Map and Network: data visualization and a globe map depicting the collaborative relationships between countries.
- Authors co-citation network: a representation of the connection network between authors based on co-citations.

Fig. (**2**) depicts the research method used in this study.

## 3 RESULTS

### 3.1 Overview

#### 3.1.1 The Sample Characteristics

In this first paragraph, we will provide a summary of our sample as well as some of its characteristics such as yearly output, document kinds, and author information. Table **1** provides an overview of our final sample's basic metrics. The sample has 1550 distinct papers in total. These publications were authored and co-authored by 3801 distinct researchers, for a total of 0.4 documents per author. 403,380 references were mentioned in all, with 1834 author keywords and 5010 keywords plus (additional keywords). The 1550 documents were published in 388 different sources and garnered an average of 15.87 citations. Around 31.43% of the 1550 multi-authored publications were created in collaboration with an international team. The vast bulk of research has been published in the previous 8.5 years.

#### 3.1.2 Scientific Production

Fig. (**3**) depicts the annual scientific output in the field of ML and DL-based brain disorder detection and diagnosis. With 4 relevant publications released in 2015, the number of documents published in 2022 has reached 433 articles. Till May 2023 the number of relevant publications is 100.

#### 3.1.3 Average Citation per Year

Fig. (**4**) depicts the annual average number of publication citations. The average number of citations per publication published in 2015 is 2.14 with 9 citable years whereas the average number of citations per publication published in 2022 is 1.43 with 2 citable years. The publications of the year 2017 have gained 12.14 average citations with 7 citable years. Up to May 2023, the average number of citations per publication published is 0.42.

#### 3.1.4 Three Field Plot: The Relationship between Authors, Countries, and Titles

**Fig. (5):**
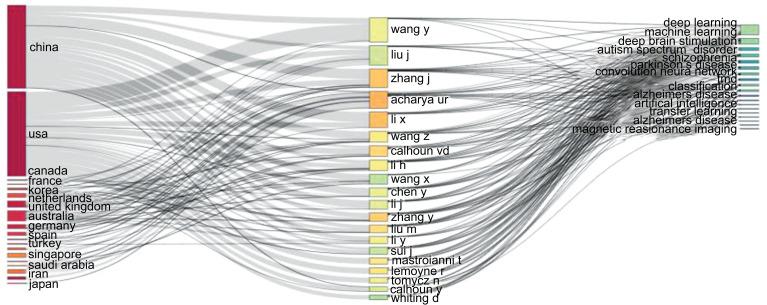
Three field plot.

The three-field figure depicts the link between the countries (left column in Fig. **5**), authors (middle column in Fig. **5**), and keywords (right column in Fig. **5**) of the relevant publications. Rectangles in various colors were used to depict the diagram's relevant components. The total of the associations forming between the elements that the rectangle represented determined the height of the rectangles. The thickness of the linkages indicates that a significant quantity of information is traveling between a group of values. The most significant study themes on ML and DL-based brain disorder detection and diagnosis have been authored by authors from China, and USA, as illustrated in Fig. (**5**). The total number of items considered is 20.

### 3.2 Sources

#### 3.2.1 The Most Productive and Top-cited Sources

Fig. (**6**) shows the top 10 most productive sources for ML and DL-based brain disorder detection and diagnosis publications. Lecture notes in computer science are the most prolific source with 37 documents, Frontiers in neuroscience journal has published 23 documents, and Biomedical signal processing and control journal has 18 documents, according to the research.

The top ten most-cited sources for works in ML and DL-based brain disorder detection and diagnosis are shown in Fig. (**7**). The Neuroimage journal was the most cited source, with 1112 citations, followed by Frontiers in neuroscience journal with 504 citations, and Neuroimage: Clinical journal came in third with 498 citations.

#### 3.2.2 Sources based on Bradford’s Law

Fig. (**8**) represents the spread of sources in the field of ML and DL-based brain disorder detection and diagnosis based on Bradford's law.

#### 3.2.3 Source’s Production Over Time

Fig. (**9**) shows the five most productive sources over time in the field of ML and DL-based brain disorder detection and diagnosis. From the illustration, we can see that Lecture notes in computer science are publishing more papers in this field over time followed by Frontiers in neuroscience journal, NeuroImage, and IEEE journal of biomedical and health informatics.

### 3.3 Authors

#### 3.3.1 The Most Relevant Authors

Fig. (**10**) depicts the ten most relevant authors. In the topic of ML and DL-based brain disorder detection and diagnosis, 3801 authors published 1550 publications. The most productive authors were Acharya, U.R. with 16 publications, with a fractionalized value of 2.63 and 457 total citations since 2018, respectively, followed by Zhang and Li with 14 publications but 530 total citations and 13 publications with 167 total citations and fractionalized values of 1.94 and 1.71 correspondingly since 2017. Calhoun V.D. is in the fourth position with 11 publications but with 858 total citations since 2016.

#### 3.3.2 The Most Relevant Institutions

Fig. (**11**) depicts the evaluation of the publishing output of organizations or author affiliations that contributed to the field of ML and DL-based brain disorder detection and diagnosis. Stanford University earned the top place with 67 articles.

#### 3.3.3 The Most Relevant Countries

As demonstrated in Table **2** and Fig. (**12**), this study also considered the countries where the authors published their contributions to the field of ML and DL-based brain disorder detection and diagnosis. The USA took first place with 94 single-country publications, 34 multi-country publications, and a frequency of 0.149. The USA, China, and India are the top three scientific productivity countries in the world in this field.

#### 3.3.4 The Most Cited Authors

Local citations indicate how often a researcher in this selection has been cited in other articles in the selection. Local citation score and global citation score measures were utilized to conduct a more in-depth evaluation of the source publications. The local citation score measured the frequency with which other publications in the collection acknowledged the authors' works in the Scopus database. Total citations reflect the number of times the articles in this collection have been referenced, according to the global citation score. However, the articles cited did not necessarily deal with ML and DL-based brain disorder detection and diagnosis. The higher the score for local citations, the more relevant the item was to the domain. The research also used bibliometrics to examine the publication output of the subject's most widely read authors. Duncan, J.S., Dvornek, N.C., Li, X., Papademetris, X., Staib, L.H., Ventola, P., and Zhuang, J. have ranked first, as shown in Fig. (**13**), with 7 citations.

Taking into account the authors’ local impact, we identified the 10 most globally cited authors in terms of total publications (TP), total citations (TC), and publication year start (PY_start), as shown in Table **3**.

#### 3.3.5 Author Productivity through Lotka's Law

This bibliometric study computes Lotka's law coefficients for ML and DL-based brain disorder detection and diagnosis articles. Lotka’s law describes the productivity of authors in a certain subject area. Lotka’s law states that “as the frequency of publications increases, the approximate number of authors with that frequency of publications may be predicted”, and in particular, “at a higher level of productivity, there are fewer authors”. This law enables the identification of the most significant authors in a certain area [31]. Lotka's Law's frequency distribution of scientific output is seen in Fig. (**14**). It can be noticed how the percentage of authors with a lower number of articles produced is very high concerning the percentage of authors with more than three documents published. Table **4** demonstrates how the number of publications and the frequency of authors in the area under examination significantly follow Lotka's law.

#### 3.3.6 Most Cited Countries

Fig. (**15**) shows the most cited countries in the field of ML and DL-based brain disorder detection and diagnosis. As per the representation, the USA has gained the maximum citation with 5595 citations followed by China with 2700 and Brazil with 1330 citations.

### 3.4 Documents

#### 3.4.1 The Most Cited Documents

The amount of citations a given document has received from any other publication in the whole Scopus Core Collection is referred to as its global citation count. Fig. (**16**) depicts the most widely referenced documents worldwide.

Arbabshirani *et al*s research “Single subject prediction of brain disorders in neuroimaging: Promises and pitfalls” [32] was published in Neuroimage Journal in 2017, rated #1 with 522 citations. This paper summarises and discusses detailed information about schizophrenia, mild cognitive impairment, Alzheimer's disease, depressive disorders, autism spectrum disorder, and attention deficit hyperactivity disorder, such as sample size, type and number of extracted features, and reported accuracy. The authors describe key weaknesses of existing research from the perspective of ML and DL. Common prejudices are explored, and recommendations are made. There is also a discussion of upcoming themes such as decentralized data sharing, multimodal brain imaging, differential diagnosis, disease subtype categorization, and deep learning.

The second most cited article, “Identification of autism spectrum disorder using deep learning and the ABIDE dataset” [33] was published in 2018 by Heinsfeld, A.S. et al in Neuroimage: Clinical journal and has been cited 462 times in the Scopus database, with an annual citation rate of 77. Using ABIDE data, the authors used DL to classify autism spectrum disorder (ASD) from controls. The authors looked at functional connection patterns that objectively identify ASD individuals in functional brain imaging data, and they sought to uncover the neural patterns that came from the classification. rs-fMRI was used to extract brain function patterns, which revealed anterior-posterior underconnectivity in the autistic brain.

Vieira *et al*. published the third most cited article “Using deep learning to investigate the neuroimaging correlates of psychiatric and neurological disorders: Methods and applications” [34] in the journal Neuroscience & Biobehavioral Reviews in 2017. This article has been referenced 365 times. The authors explained the fundamental ideas of DL and reviewed research that employed this technique to categorize brain-based disorders in this paper. This article discusses future research and the issues of DL in neuroimaging.

Habes *et al.* published “White matter hyperintensities and imaging patterns of brain aging in the general population” [35] in Brain journal in 2016 which is the fourth most cited article. This study looks at the association between the burden of white matter hyperintensities and patterns of brain shrinkage associated with brain aging and Alzheimer's disease in a large population-based sample (n = 2367) with a wide age range (20-90 years) from the Pomerania Health Study.

Another work of the year 2016 was the fifth most cited document, “Deep neural network with weight sparsity control and pre-training extracts hierarchical features and enhances classification performance: Evidence from whole-brain resting-state functional connectivity patterns of schizophrenia” [36] published by Kim *et al*. in NeuroImage journal. The goal of this study was to use a deep neural network (DNN) to classify whole-brain resting-state Functional connectivity (FC) patterns in schizophrenia (SZ) patients *vs.* healthy controls (HCs) and to identify aberrant FC patterns associated with SZ.

The sixth most cited (211) article is entitled “Brain MRI Analysis for Alzheimer’s disease diagnosis using an ensemble system of deep convolutional neural networks” [37] and was published by Islam *et al*. in Brain Informatics journal in 2018. Using brain MRI data analysis, the authors proposed a deep convolutional neural network for Alzheimer's disease diagnosis and identifying distinct phases of Alzheimer's disease.

Likewise, “A Review on a Deep Learning Perspective in Brain Cancer Classification” [38] was the seventh most cited (198) document. Tandel *et al.* published this study in the Cancers journal in 2019. The authors summarize the pathophysiology of brain cancer, imaging modalities for brain cancer, and automatic computer-aided approaches for brain cancer characterization in an ML and DL paradigm in this study. Another goal of this article was to identify present problems with existing engineering approaches and to forecast a future paradigm. Furthermore, in the context of machine learning and the deep learning paradigm, the authors have emphasized the association between brain cancer and other brain disorders such as stroke, Alzheimer's, Parkinson's, Wilson's disease, leukoaraiosis, *etc*.

“Hierarchical Fully Convolutional Network for Joint Atrophy Localization and Alzheimer's Disease Diagnosis Using Structural MRI” [39], the eighth paper, was published in 2020 and has been referenced 190 times in the Scopus database, with a citation rate of 47.5 each year. Lian *et al.* published this paper in IEEE Transactions on Pattern Analysis and Machine Intelligence journal. The authors propose a hierarchical fully convolutional network (H-FCN) to automatically identify discriminative local patches and regions in whole brain structural magnetic resonance imaging, which is then used to jointly learn and fuse multi-scale feature representations to construct hierarchical classification models for Alzheimer's disease diagnosis.

The ninth most-cited paper (183 citations) is titled “3D CNN Based Automatic Diagnosis of Attention Deficit Hyperactivity Disorder Using Functional and Structural MRI” [40] and was published in IEEE Access magazine in 2017 by Zou *et al.* The authors provide a DL-based attention deficit hyperactivity disorder classification approach using 3-D convolutional neural networks (CNNs) applied to magnetic resonance imaging data in this research.

Finally, Graham *et al*. published “Artificial Intelligence for Mental Health and Mental Illnesses: an Overview” [41] (158 citations) in Current Psychiatry Reports in 2019 with 31.6 citations each year. This article provides an overview of artificial intelligence (AI) and its current applications in healthcare, a review of recent original research on AI specific to mental health, and a discussion of how AI can supplement clinical practice while taking into account its current limitations, areas for further research, and ethical implications regarding AI technology.

The number of citations a certain document has received from any other article in the researched dataset is known as its local citation count, in our instance, the area of ML and DL-based brain disorder detection and diagnosis. Fig. (**17**) depicts the most frequently mentioned documents on a local level.

LI *et al.* paper “2-Channel convolutional 3D deep neural network (2CC3D) for fMRI analysis: ASD classification and feature learning” [42] was ranked #1 with 7 local and 49 global citations. This article was published in the IEEE 15th International Symposium on Biomedical Imaging (ISBI 2018) in 2018. The authors suggest a new whole-brain fMRI-analysis technique to diagnose autism spectrum disorder (ASD) and investigate biological markers in ASD classification in this research.

The second most locally cited article, “Synthetic structural magnetic resonance image generator improves deep learning prediction of schizophrenia” [43] was published in 2015 by Ulloa *et al*. in the IEEE 25^th^ International Workshop on Machine Learning for Signal Processing (MLSP) and has been locally cited twice with 21 global citation times in the Scopus database. The authors of this article employed a feed-forward neural network to classify schizophrenia patients and healthy controls using structural magnetic resonance data.

Khan *et al.* published the third most locally cited article “A deep learning based scoring system for prioritizing susceptibility variants for mental disorders” [44] in the IEEE International Conference on Bioinformatics and Biomedicine (BIBM) in 2017, with 2 and 4 local and global citations in the Scopus database. The authors created a computational tool, a deep learning-based scoring system (ncDeepBrain), to analyze whole genome/exome sequencing data on personal genomes by incorporating contributions from coding, non-coding, structural variants, known brain expression quantitative trait loci (eQTLs), and PsychENCODE enhancer/promoter peaks.

In 2018, Yao *et al.* published “Resting Tremor Detection in Parkinson's Disease with Machine Learning and Kalman Filtering” [45] at the IEEE Biomedical Circuits and Systems Conference (BioCAS), which is the fourth most locally cited (2) paper with 28 global citations. The authors suggest using an ML technique to detect resting-state tremors using local field potentials (LFPs) collected from the subthalamic nucleus (STN) in 12 Parkinson's patients.

Yang *et al.* published “Functional connectivity magnetic resonance imaging classification of autism spectrum disorder using the multisite ABIDE dataset” [46] in the IEEE EMBS International Conference on Biomedical & Health Informatics (BHI) in 2019 which is the fifth most locally cited document with 24 global citations. The goal of this paper is to use ML algorithms to classify ASD patients and typically developing (TD) participants using resting-state functional MRI (rs-fMRI) data from the ABIDE (Autism Brain Imaging Data Exchange) large multisite data repository and identify important brain connectivity features.

The sixth locally cited (2) article entitled “A CNN Model: Earlier Diagnosis and Classification of Alzheimer Disease using MRI” [47] was published by Salehi, A.W. et al in the International Conference on Smart Electronics and Communication (ICOSEC) in 2020 with 38 global citations. In this article, the authors employed MRI pictures, the ADNI 3 class of images, and a total of 1512 mild, 2633 normal, and 2480 AD to develop a Convolutional Neural Network (CNN) for the early identification and categorization of AD.

In contrast, “Implementation of a smartphone wireless accelerometer platform for establishing deep brain stimulation treatment efficacy of essential tremor with machine learning” [48] was the seventh most locally cited (1) document with 32 global citations. LeMoyne *et al.* published this study at the 37^th^ Annual International Conference of the IEEE Engineering in Medicine and Biology Society (EMBC) in 2015. In this study, authors used deep brain stimulation for the diagnosis of essential tremors.

“Brain MRI analysis for Alzheimer’s disease diagnosis using an ensemble system of deep convolutional neural networks” [37], the eighth paper, was published in 2018 and has been globally referenced (sixth position) 211 times in the Scopus database, with a local citation 1. Islam *et al.* published this paper in Brain Informatics journal.

The ninth most locally cited paper (1 citation) with 4 global citations is titled “On the need for adaptive learning in on-demand Deep Brain Stimulation for Movement Disorders” [49] and was published in the 40^th^ Annual International Conference of the IEEE Engineering in Medicine and Biology Society (EMBC) in 2018 by Khobragade *et al.* In this study, two ML algorithms-Decision Tree and Large Memory STorage And Retrieval (LAMSTAR) neural networks-with surface Electromyography and accelerometry as control signals--are used to predict the onset of tremor after Deep Brain Stimulation (DBS) turned off in two patients, one with Parkinson's disease and the other with essential tremor.

Finally, Choi *et al*. published “Deep learning only by normal brain PET identify unheralded brain anomalies” [50] (1 local and 34 global citations) in eBioMedicine journal in 2019. In this article, the Abnormality Score was defined as how much a particular brain picture deviates from normal data using variational autoencoder which is a sort of unsupervised learning.

#### 3.4.2 The Most Frequent Words

The words “deep learning” and “machine learning” were the most frequently used by authors, with a total of 300 and 213 occurrences, followed by “eeg” and “classification” with 53 and 51 occurrences. Fig. (**18**) depicts the most frequently used terms in the field of ML and DL-based brain disorder detection and diagnosis. Fig. (**19**) depicts a word cloud of the most commonly used terms in the study on the subject under consideration. From the depiction, we can say that magnetic resonance imaging is the most popular modality for ML and DL-based brain disorder detection and diagnosis.

#### 3.4.3 Word’s Frequency Over Time

Fig. (**20**) shows the word’s frequency over time as per keyword plus in the field of ML and DL-based brain disorder detection and diagnosis for the period January 2015 - May 2023. From the representation, we can see that the frequency of using the terms “deep learning”, “human”, “female”, “brain”, “electroencephalography” *etc*. increasing over time.

#### 3.4.4 Trend Topics

Fig. (**21**) depicts the trend topics in the field of ML and DL-based brain disorder detection and diagnosis. Till May’23 the topic “biomarkers”, “t1 weighted imaging” and “tinnitus” were the hot topics with frequencies 24, 10, and 9 respectively. In 2022, the topics “electroencephalography”, “convolutional neural network” and “procedures” were the hot topics with frequencies 296, 235, and 141 respectively. In 2021 the topics “deep learning”, “brain” and “human” were the hot topics with frequency 688, 475, and 471 respectively.

#### 3.4.5 Most Locally Cited References

The number of citations a certain reference has received from any other article in the researched dataset is known as its local cited reference, in our instance, the area of ML and DL-based brain disorder detection and diagnosis. Table **5** depicts top ten the most cited references on a local level. Lecun *et al’s* publication “Deep learning” [51] published in 2015 in Nature Journal placed first with 49 citations. In this article, the authors have discussed different DL-based techniques available that can be applied in the medical field. The second most referenced (25) citation is He *et al's* work “Deep Residual Learning for Image Recognition” [52] published in Proceedings of the IEEE Conference on Computer Vision and Pattern Recognition (CVPR) in 2016. The authors give extensive empirical data in this study demonstrating that residual networks are easy to optimize and can gain accuracy from much greater depth. The book “Deep Learning” [53] was published by Goodfellow *et al*. in MIT Press in 2016 and gained the third position (23 citations) in terms of the most cited reference. In this book, authors have covered linear algebra, probability, and information theory, different numerical computations, ML basics, deep feedforward networks, regularization for deep learning, optimization for training deep models, convolutional networks, recurrent networks, recursive networks, different applications, autoencoders, linear factor models, monte carlo method, deep generative models, *etc*.

### 3.5 Conceptual Structure

#### 3.5.1 Keyword Co-occurrence Network

Fig. (**22**) depicts the keyword co-occurrence network. Each node in the network represents a keyword. The size of the node indicates the frequency of the keyword (how frequently the term appears). The link between the nodes represents the co-occurrence of terms (terms that appear together or co-occur), and the thickness of the association shows the frequency with which two or more terms appear together. Each color represents a topic grouping. There are 6 clusters. Cluster 1 (red) includes 31 terms such as “deep learning”, “machine learning”, “Alzheimer’s disease”, “neuroimaging”, “epilepsy”, “MRI”, “schizophrenia” *etc*. Cluster 2 (blue) consists of only two keywords such as “electroencephalogram (EEG)” and convolutional neural network (CNN). Cluster 3 (green) includes 11 keywords like “autism spectrum disorder”, “fMRI”, “deep neural network”, “functional connectivity” *etc*. Cluster 4 (violet) consists of only 4 keywords such as “deep brain stimulation”, “Parkinson's disease”, “Parkinson’s disease” and “essential tremor”. Cluster 5 (orange) and cluster 6 (brown) include only one keyword “machine learning (ml)” and “magnetic resonance imaging (MRI)” respectively.

#### 3.5.2 Thematic Map

Thematic maps are keyword clusters that may be grouped into only one circle and mapped as a two-dimensional representation utilizing the density and centrality of their keywords [59-61]. As shown in Fig. (**23**), a thematic map is split into quadrants based on their location. Niche topics appear in the upper-left quadrant; they are keywords with a high degree of development but may not be extremely important. In our sample, “deep brain stimulation”, “major depressive disorder”, and “machine learning” fall into this category. The lower-left quadrant contains emerging or diminishing subjects, which are keywords of low to medium significance and development degrees. In this category “convolutional neural network”, “magnetic resonance imaging” and “functional connectivity” are there as per our data. There are motor themes in the upper-right quadrant with the highest level of development and importance. In this category “deep learning”, “machine learning”, “EEG”, “essential tremor”, “neuroimaging”, “autism spectral disorder” *etc* are there. The lower-right quadrant contains basic themes, which are terms with a high relevance degree and a low to medium development degree. In this category “epilepsy”, “deep neural network”, “Parkinson’s disease”, “transfer learning”, “dementia”, “support vector machine” *etc* are there. We generated the thematic map using the “author keyword” and “walktrap” clustering algorithms.

#### 3.5.3 Factorial Analysis

Figs. (**23** and **24**) represent the factorial analysis using multiple correspondence analysis (MCA) and correspondence analysis (CA). MCA [62-64] is used to visually and statistically analyze multivariate categorical data. It explores the interplay of a group of categorical data to find new latent variables or components. The relative placements and distribution of the dots along the dimensions are used to interpret the results; the closer the words are put, the more similar the distribution is. Fig. (**24**) shows how the ML and DL-based brain disorder detection and diagnosis publications were analyzed using 30 author keywords and then ranked based on their overall citation count. There are two groups of keywords: Cluster 1 (28 keywords) and Cluster 2 (2 keywords). The densest cluster is represented in red color in Fig. (**23**). This category contains the vast majority of accepted and published new research. This cluster is quite powerful. The bulk of publications in this field contains study subjects including “convolutional neural network”, “transfer learning”, “Parkinson's disease”, “magnetic resonance imaging”, “depression”, “neuroimaging”, “machine learning”, “epilepsy”, “deep learning” *etc*. Cluster 2 (represented in blue) indicates the learning algorithms and systems for forecasting. This cluster includes two terms: “Parkinson's disease” and “deep brain simulation”.

CA [65-67] is a visual way of studying how the variables in a contingency table relate to one another. It provides a way to reduce and describe data sets using two-dimensional graphs. The goal, as seen in Fig. (**24**) is to produce a full data picture that may be utilized for interpretation. Fig. (**24**) was created by using 30 author keywords and then ranking them based on their overall citation count. We discovered two keyword clusters: Cluster 1 (36 keywords) and Cluster 2 (4 keywords) and two publication clusters: Cluster 1 (593 publications) and cluster 2 (12 publications). The red area in Fig. (**25**) indicates that most of the research has been done on these topics including “autism spectral disorder”, “Parkinson's disease”, “schizophrenia”, “electroencephalogram”, “electroencephalography” *etc*.

### 3.6 Network Analysis

#### 3.6.1 Country Collaboration Network

As seen in Figs. (**26** and **27**), the USA is at the heart of the largest cluster of cooperation, followed by China, the United Kingdom, Brazil, and Canada. Another cluster is visible between Germany, Australia, India, Netherlands, Italy, and Egypt. There will also be a third between Saudi Arabia, Spain, Iran, Korea, Singapore, and France.

#### 3.6.2 Author Co-citation Network

Fig. (**28**) depicts a network of author co-citations. The network is organized into four clusters that reflect author co-citations. The most referenced author in Cluster 1 (red) is Acharya, U.R. Calhoun, V.D. the author with the most citations in Cluster 2 (blue). In Cluster 3 (green), Shen, D. is the most referenced author. In Cluster 4 (yellow), Dvornek, N.C. and Craddock, R.C. is the most mentioned author.

## 4 DISCUSSION AND FUTURE RESEARCH AGENDA

The findings of this bibliometric study and content research have led to several views and implications that have stirred much controversy. This study included 1550 publications on ML and DL-based brain disorder detection and diagnosis authored by 3801 authors between 2015 and May 2023. The annual growth rate is 36.78% and the average citation rate is 15.87. According to the numbers, the majority of documents were co-written, with just 22 being single-authored, indicating a very high degree of collaboration in this area.

The three-field plots, which use three essential metadata fields, provide useful insights into the relationship between domains, such as relating authors' work to particular keywords and nations participating in the study area. The most significant contributions to the research of ML and DL-based brain disorder detection and diagnosis were made by Acharya, U.R. from Singapore, Zhang, J. from China, as well as Calhoun VD from the USA in terms of citation impact. Our data indicate a tight relationship between topic studies in the USA, China, and India.

Fig. (**6**) revealed that the conference proceedings series Lecture Notes in Computer Science (LNCS) [68] provides the most recent academic breakthroughs in all fields of computer science. LNCS volumes, along with its subseries Lecture Notes in Artificial Intelligence (LNAI) [69] and Lecture Notes in Bioinformatics (LNBI) [70], are submitted for indexing in the Conference Proceedings Citation Index (CPCI), which is part of Clarivate Analytics' Web of Science; Scopus; EI Engineering Index; Google Scholar; DBLP; and other databases.

Frontiers in Neuroscience journal [71] is a peer-reviewed publication that publishes thoroughly peer-reviewed material from a wide range of professions and fields. This open-access publication is at the forefront of distributing and communicating scientific information and groundbreaking discoveries to researchers, universities, doctors, and the general public throughout the world.

The goal of the Biomedical Signal Processing and Control journal [72] is to create an international cross-disciplinary platform for the exchange of information on research in the measurement and analysis of signals and pictures in clinical medicine and the biological sciences. Contributions dealing with practical, applications-led research on the utilization of methodologies and technology in clinical diagnosis, patient monitoring, and management are prioritized. This publication highlights the primary areas in which these technologies are applied and developed at the intersection of engineering and clinical science.

Fig. (**7**) reveals that NeuroImage [73], a Journal of Brain Function, serves as a platform for conveying significant breakthroughs in the use of neuroimaging to investigate structure-function and brain-behavior links. Though the emphasis is on the macroscopic level of human brain organization, breakthroughs in meso-and microscopic neuroimaging across all species are published in this journal if they contribute to a systems-level knowledge of the human brain. The key criterion used to evaluate publications for NeuroImage is the extent to which the scientific contribution advances our understanding of brain function, organization, and structure.

Bradford's Law investigates the aggregation of papers in a certain field inside a specific set or zone of academic publishing journals and deals with journal efficiency in terms of quantitative publications as seen in Fig. (**8**). Bradford's Law of Spread Publications argues that a large number of publications are spread among a small number of journals, while a large number of journals have a correspondingly smaller number of articles. Articles scattered throughout many periodicals can be separated into an approximated percentage. Bradford's law of scattering states that writings on a specific subject are distributed in roughly a set mathematical ratio.

Fig. (**9**) shows original papers highlighting contemporary breakthroughs in the field of biomedical and health informatics where information and communication technologies interface with health, healthcare, life sciences, and biomedicine and are published in the IEEE Journal of Biomedical and Health Informatics [74]. This includes, but is not limited to, the acquisition, transmission, storage, retrieval, management, processing, and analysis of biomedical and health information; applications of information and communication technologies in healthcare, public health, patient monitoring, preventive care, early disease diagnosis, the discovery of new therapies, and patient-specific treatment protocols leading to improved outcomes.

The analyses also revealed four clusters of keywords, each with one or more terms. Cluster 1 (red) includes 31 terms such as “deep learning”, “machine learning”, “Alzheimer’s disease”, “neuroimaging”, “epilepsy”, “MRI”, “schizophrenia” *etc*. Cluster 2 (blue) consists of only two keywords such as “electroencephalogram (EEG)” and convolutional neural network (CNN). Cluster 3 (green) includes 11 keywords like “autism spectrum disorder”, “fMRI”, “deep neural network”, “functional connectivity” *etc*. Cluster 4 (violet) consists of only 4 keywords such as “deep brain stimulation”, “Parkinson's disease”, “Parkinson’s disease” and “essential tremor”. Cluster 5 (orange) and cluster 6 (brown) include only one keyword “machine learning (ml)” and “magnetic resonance imaging (MRI)” respectively. These findings reflect the extensive usage of ML and DL for brain disorder detection and diagnosis and their application to psychiatry using MRI (cluster 1), specific application to Autism (cluster 3) possibly driven by the early availability of a large public database of fMRI data from Autistic subjects in the form of ABIDE database, the application of Parkinson’s and the widespread clinical use of deep brain stimulation for Parkinson’s (cluster 4) and the generic use of EEG and MRI data to drive ML and DL algorithms for brain diagnosis (rest of the clusters). This also indicates that EEG and MRI modalities dominate the field despite the clinical use of other modalities (such as PET/SPECT, CT, fNIRS, *etc*).

Using knowledge frameworks, thematic maps represent the structural and dynamic components of a study domain. They were utilized to develop conceptual structures that identified major topics, subjects, and intellectual frameworks that classified how an author's work affected this scientific community. These conceptual structures served to offer a comprehensive overview of the major developments and discoveries in ML and DL-based brain disorder detection and diagnostic research. Another application may be the study of how ideas or conditions evolve throughout time. This method provides academics with a list of the most well-known articles for each subject cluster, which may be used to focus research on a certain theme. The scientific map can provide statistics on the relevance of topics based on centrality and density, allowing for projections of possible future growth. From the thematic map discussed in this study, it can be shown that “machine learning”, “deep brain stimulation” *etc* have a high degree of development. Medium significance is on “convolutional neural network”, “MRI” *etc*. The highest level of development in this field is on “deep learning”, “EEG” *etc* whereas the high relevance degree is on “deep neural network”, “transfer learning” *etc*.

The results of our bibliometric research demonstrate that the most renowned publications are from a small number of authors. Because the bulk of publications are open access, contributions are promptly shared with the public, and a huge number of authors develop as the field advances. It is also worth noting that papers in the field are being increasingly referenced (with 522 citations for the most cited work), demonstrating how relevant it is right now. The USA, India, and China are the top three countries in terms of research production in this sector. These findings are not surprising considering that these countries' top Nation Rank's rankings for global scientific production across all categories. The findings reveal that even the most prolific researchers employ a diversity of methodologies and skills, confirming the field’s interdisciplinarity. Journals must be both functional and thorough to successfully communicate knowledge to all players. Our research found that “Neuroimage” and “Frontiers in neuroscience” journals have the most citations on the subject.

This study focused on papers about ML and DL-based brain disorder detection and diagnosis that were indexed in the Scopus database. While comparing datasets from many databases is beyond the scope of this study, searching them may provide distinct groups of things, and the results of this analysis may differ.

We reported the findings of our bibliometric investigation in the preceding sections. In this part, we will provide possible directions for further study based on our findings. These are intended to serve as a starting point for interested academics. It is critical to examine how the interplay of ML, DL, and humans may or should appear in the context of brain disorder detection and diagnosis. Future studies should look at which type of collaboration between ML, DL, and humans [75] is most suited for brain disorder detection and diagnosis. This raises the question of whether an ML and DL-based system in place of a physician for brain tumor detection and diagnosis is even possible or not. ML and DL have the potential to help with the case of brain disorder detection and diagnosis, improve brain MRI scanning and segmentation, support decision-making, and anticipate disease risk. As a result, ML and DL in brain disorder detection and diagnosis are utilized to aid medical scientists, lab personnel, and researchers in the health care business. ML and DL in brain disorder detection and diagnosis assist doctors in providing disease analysis and guiding them in treating a specific ailment more effectively. As a result, doctors' medical judgments may be made more prudently, and standards are increasing. Furthermore, trust between the ML and DL-based brain disorder detection and diagnostic model and the humans involved is critical. Although ML and DL algorithms frequently outperform human experts in terms of accuracy [76], there is a lack of trust in the predictions provided by these systems. It should thus be examined why there is a lack of confidence and how trust in ML and DL-based systems may be increased. This is also true for patients who may be subjected to therapies based mostly on the outcomes of these systems.

The security and robustness of the ML and DL-based models are also crucial considerations [77-79]. Many ML and DL models published in the literature have only been tested on a single dataset. As a result, it's unknown if the ML and DL models can be applied to input data from multiple scanning devices. As a result, it would be useful to examine how the ML and DL models should be developed to ensure portability. It may also make sense in this context to assess ML and DL models using several datasets supplied by various sensors or manufacturers. As previously said, ML and DL systems need a significant quantity of data to learn and construct solid models. When it comes to data, it is also critical to ensure the credibility, dependability, and security of the sources or platforms from which the data is derived. If malicious actors are successful in altering or modifying the data used as input for the ML and DL system, the outcome of the ML and DL system may be affected. As a result, these results are no longer dependable and may harm the patient's health due to the risk of incorrect outcomes. Data storage is particularly crucial since medical data is subject to strict data-protection requirements [80-82]. As a result, whether storage options have complied with legislation and how to ensure that data is not traceable should be investigated. Future studies should investigate if data masking is adequate or whether total anonymization is necessary in this circumstance. Various academics are also investigating whether emerging technologies for distributed data storage and administration, such as blockchain, are suitable for medical data. A future study might thus examine whether a blockchain makes sense for the goal of organizing and preserving medical data, or whether alternative technologies and databases are more appropriate. Furthermore, it is worth noting that there are currently a few research available that look into the security and resilience of ML and DL models for brain disorder detection and diagnosis. External validation of ML and DL algorithms and robustness testing against adversarial pictures, as well as rigorous data pre-treatment, are potential approaches for achieving robustness and security goals and should thus be researched further. In this context, design science research might be used to iteratively address specific security issues to identify an efficient solution.

Only Scopus was employed in this work to derive bibliometric datasets. Future research might compile data from numerous digital libraries, such as PubMed, and Web of Science, into a single dataset. As a result, the target dataset for the study would be significantly bigger, allowing for a far broader examination. When collecting datasets from Scopus, we used 8.5 years as a constraint in the search circumstances. A considerably greater range of study findings might be discovered by looking over 10 years of publishing. Finally, non-English materials were not removed or chosen, which aided the automatic bibliometric analysis. However, a linguistic bias might have occurred. Non-English publications might be translated by language translators, allowing them to be included in the dataset as well.

Table **6** below summarizes our future research plan and highlights some future research issues that might assist in developing the area of ML and DL for brain disorder detection and diagnosis.

## CONCLUSION

This bibliometric analysis article assessed 1550 publications from January 2015 to May 2023 in light of 16 research topics. This work examined and assessed global research productivity linked to ML and DL-based brain disorder detection and diagnosis using data from the Scopus database. We used VOSviewer and the Biblioshiny program from the Bibliometrix package for R to analyze the data and create a wonderful visualization. Scientific publishing statistics reveal an annual growth rate of 36.78%, with an average citation rate of 15.87.

The United States, China, and India were among the most influential and productive countries, with the most publications. These were also the most visible countries in the collaboration network. Institutional collaborations seemed to be the most prevalent in the United States, with China, the United Kingdom, Brazil, and Canada also ranking among the most productive countries. In terms of the number of publications, Stanford University fared the best. The United States was among the best-performing countries in terms of both collaboration and yearly publication performance. The most popular ML and DL models were support vector machines and CNNs, respectively, with transfer learning emerging as a population technique to address limited data availability in certain applications of DL.

The most common brain disorder data modalities are MRI and EEG. Some researchers also highlighted the application of fMRI for the automated identification of brain diseases. Also, ML and DL applications mostly targeted Parkinson's, Alzheimer's, autism, epilepsy, and schizophrenia. Our study also points towards potential directions for future research including explainability of models, data privacy issues, and human-AI interactions in the clinic for diagnostic decision-making. In conclusion, our study documents growing interested and productivity in this field, which may continue to grow exponentially until many limitations are sorted out and ML/DL-based diagnosis of brain disorders becomes robust enough for adoption in clinical practice.

## Figures and Tables

**Fig. (1):**
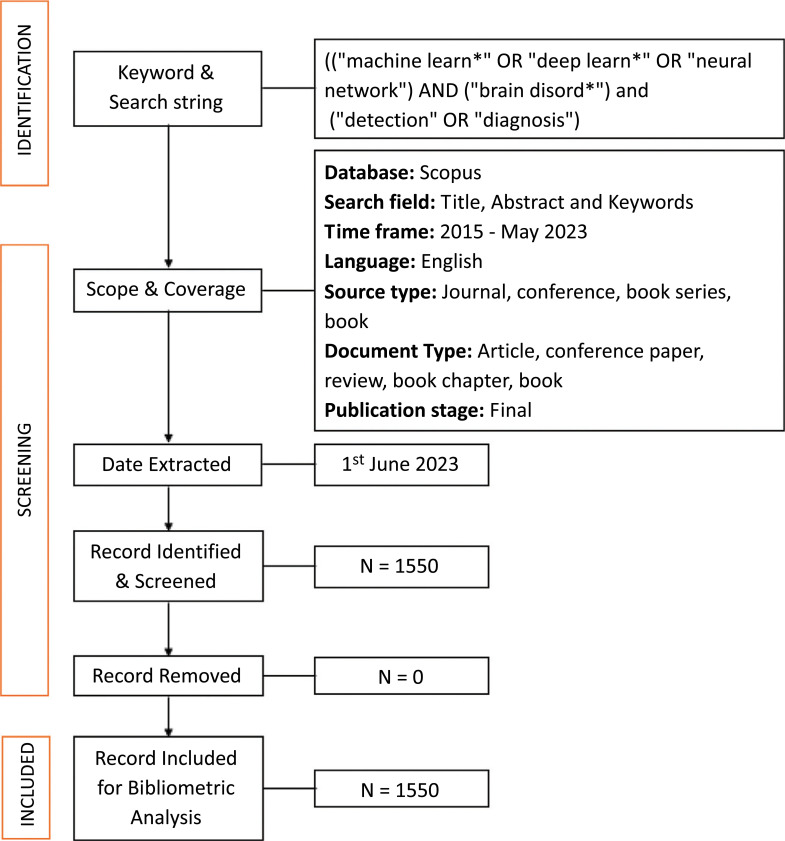
The pictorial depiction of the bibliometric data collection method.

**Fig. (2):**
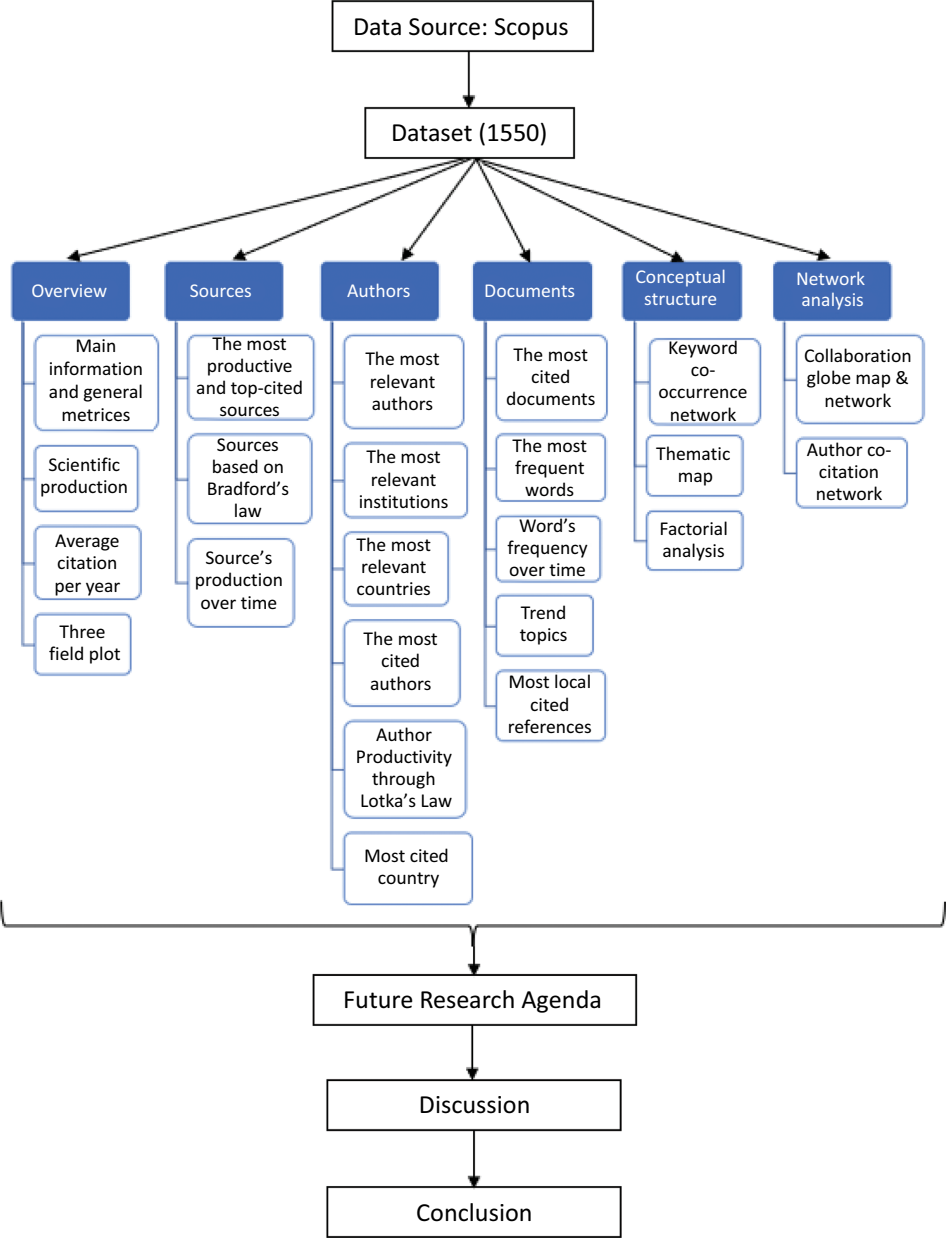
The research method used in this study.

**Fig. (3):**
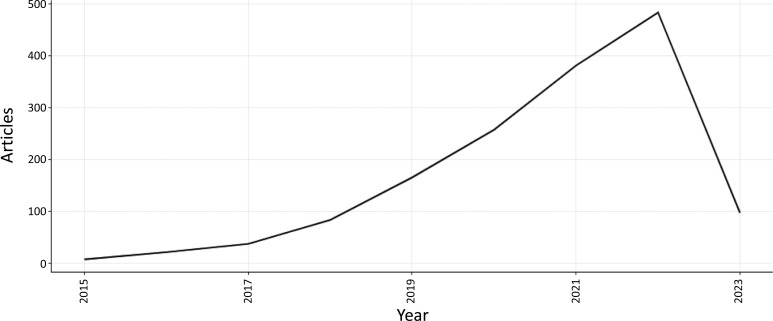
The annual scientific output.

**Fig. (4):**
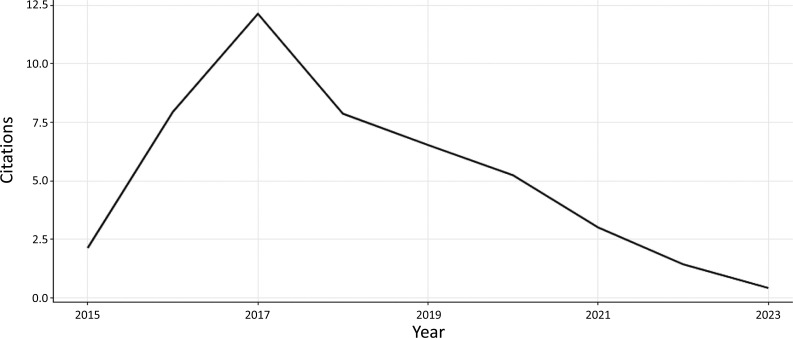
The annual average number of publication citations.

**Fig. (6):**
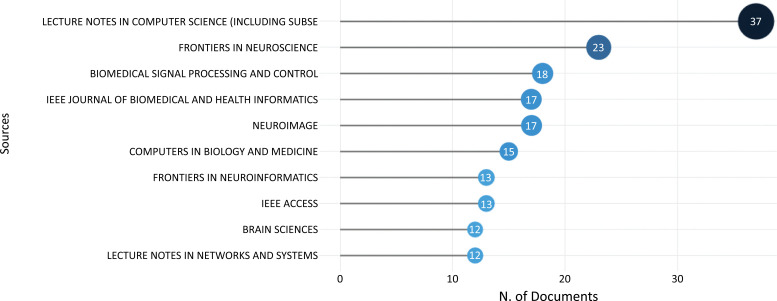
The most productive sources.

**Fig. (7):**
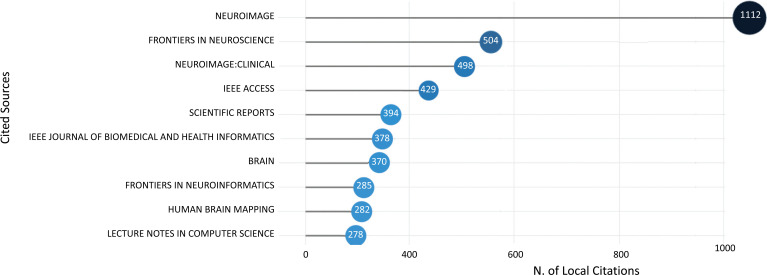
The top-cited sources.

**Fig. (8):**
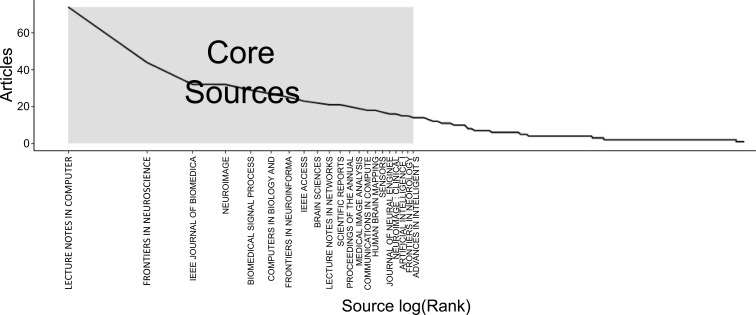
The spread of sources based on Bradford's law.

**Fig. (9):**
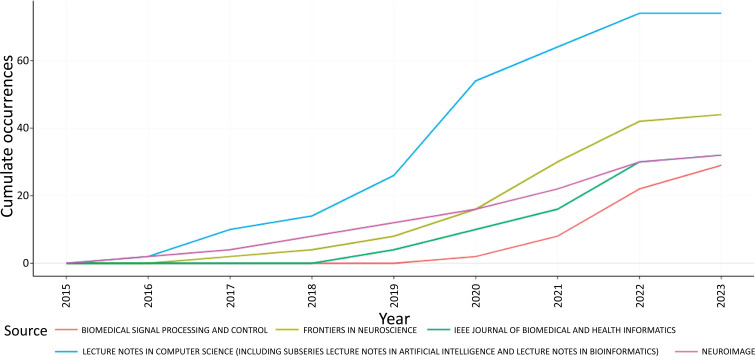
Source’s production over time.

**Fig. (10):**
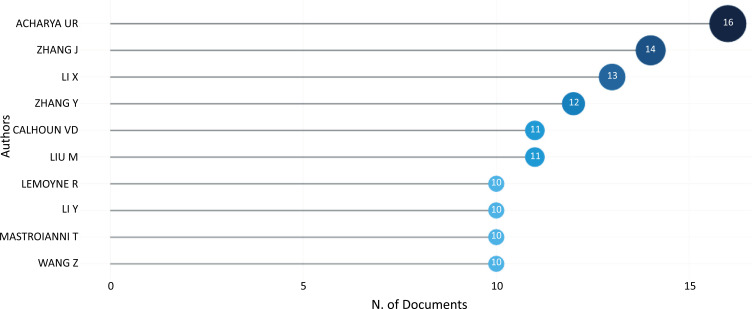
The most relevant authors.

**Fig. (11):**
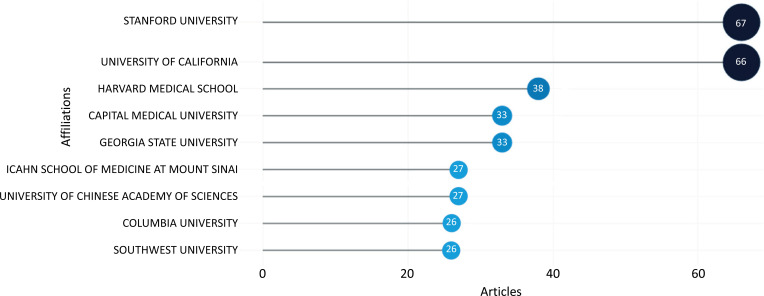
The most relevant institutions.

**Fig. (12):**
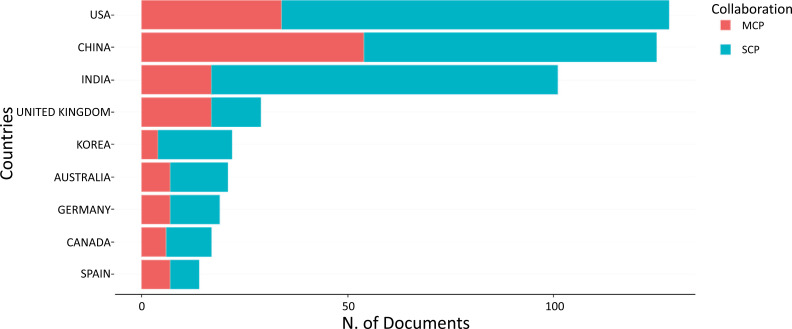
The most relevant countries by corresponding authors.

**Fig. (13):**
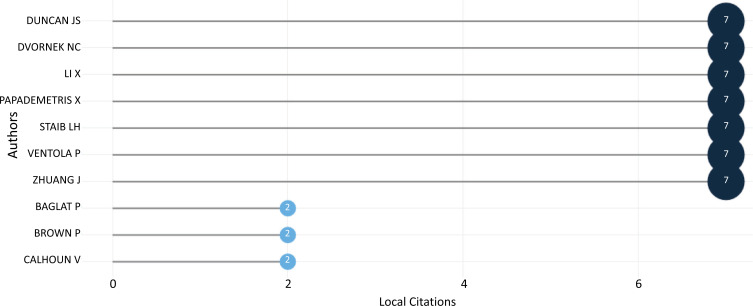
The most cited authors.

**Fig. (14):**
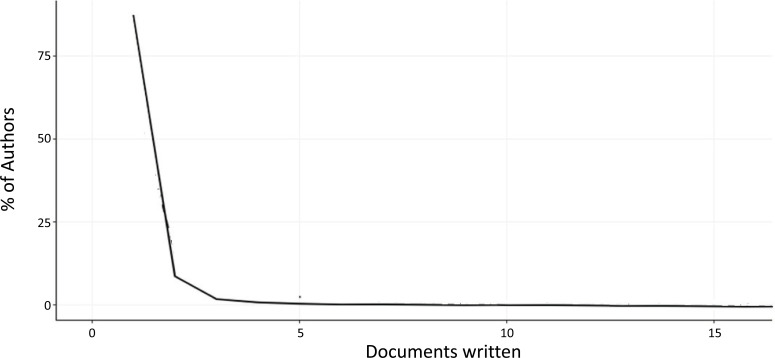
Author Productivity through Lotka’s Law.

**Fig. (15):**
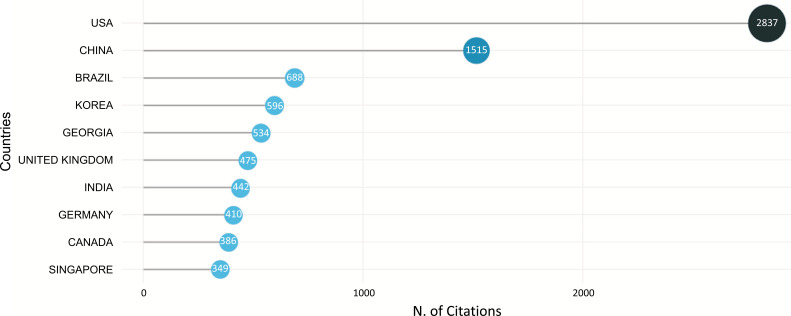
Most cited countries.

**Fig. (16):**
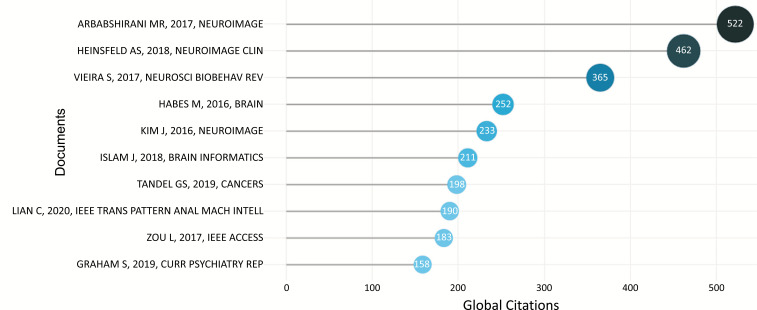
The most globally cited document.

**Fig. (17):**
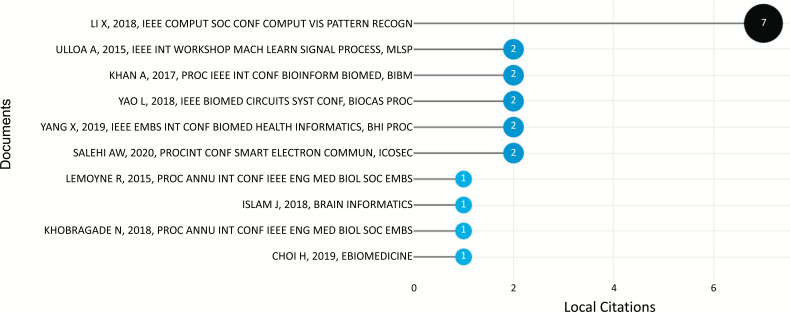
Most locally cited document.

**Fig. (18):**
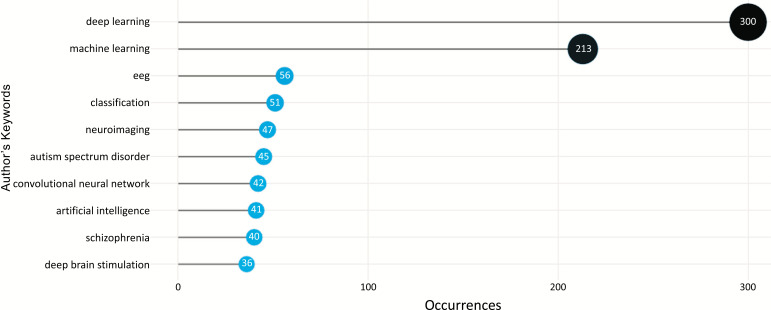
Most frequently used terms.

**Fig. (19):**
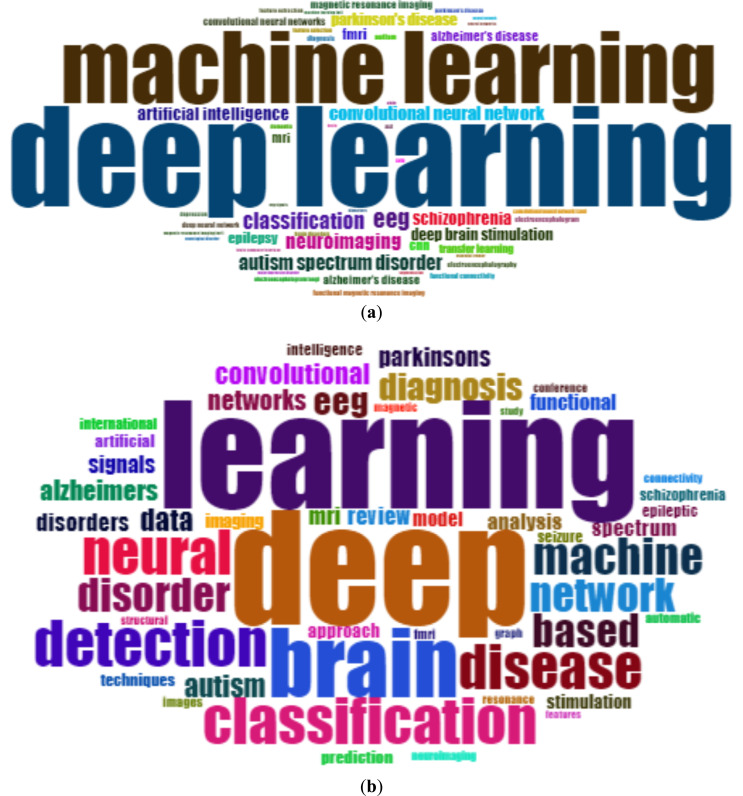
Word cloud of most frequently used terms: (**a**) based on keywords, (**b**) based on the title.

**Fig. (20):**
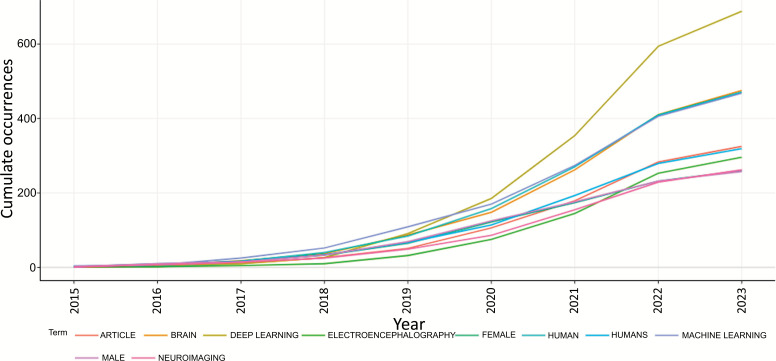
Word’s frequency over time.

**Fig. (21):**
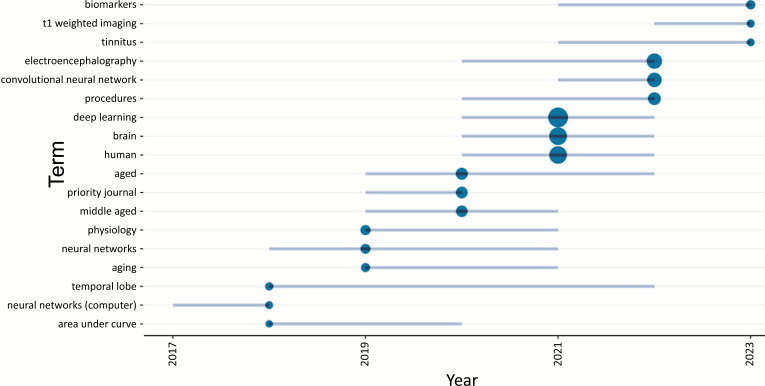
Trend topics in the field of ML and DL-based brain disorder detection and diagnosis.

**Fig. (22):**
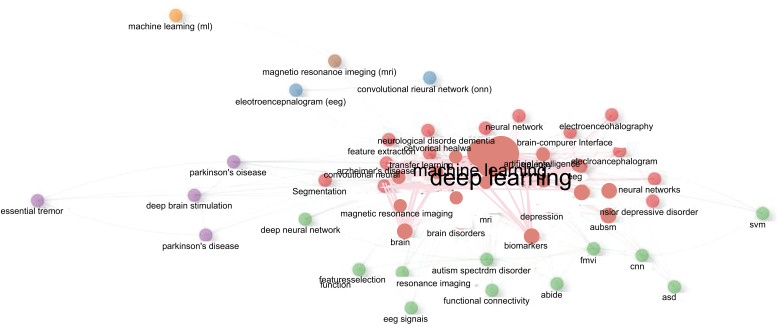
Keyword co-occurrence network.

**Fig. (23):**
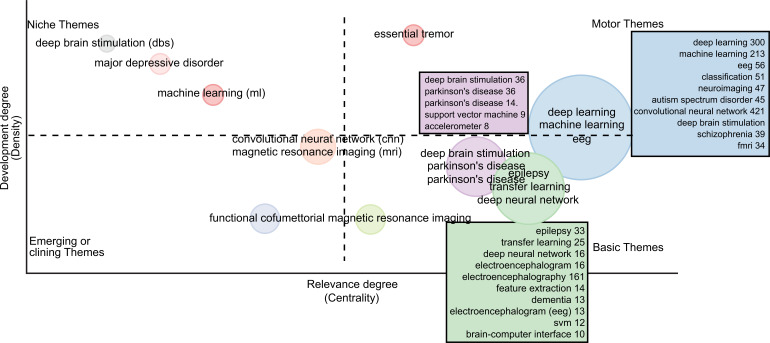
The thematic map of ML and DL-based brain disorder detection and diagnosis.

**Fig. (24):**
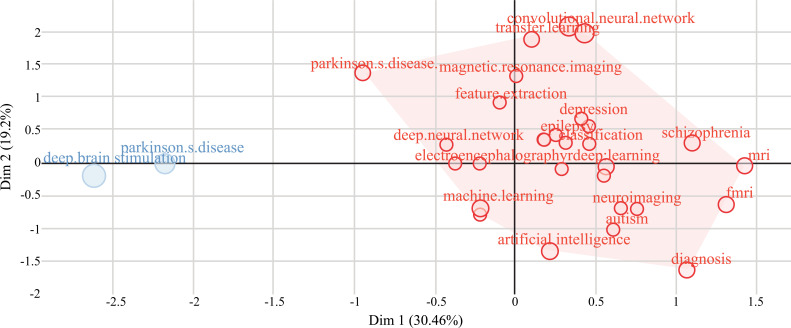
The factorial analysis using multiple correspondence analysis.

**Fig. (25):**
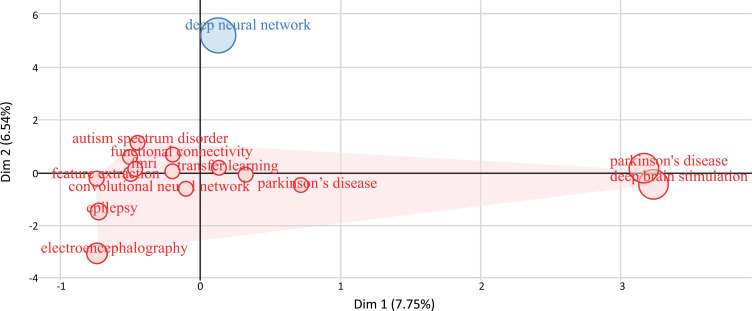
The factorial analysis using correspondence analysis.

**Fig. (26):**
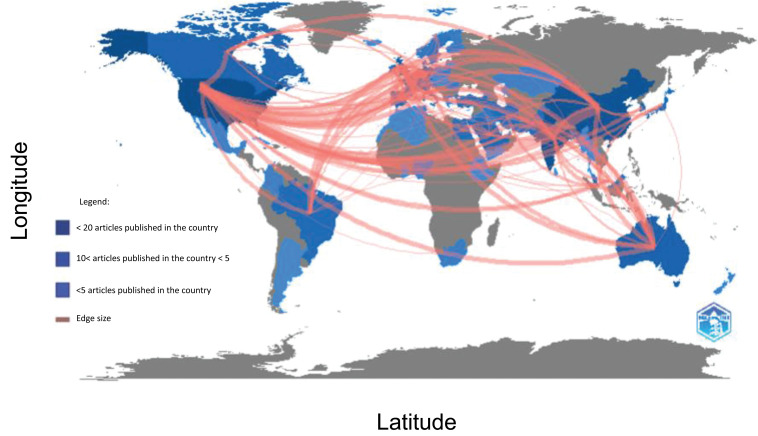
Countries’ collaboration world map.

**Fig (27):**
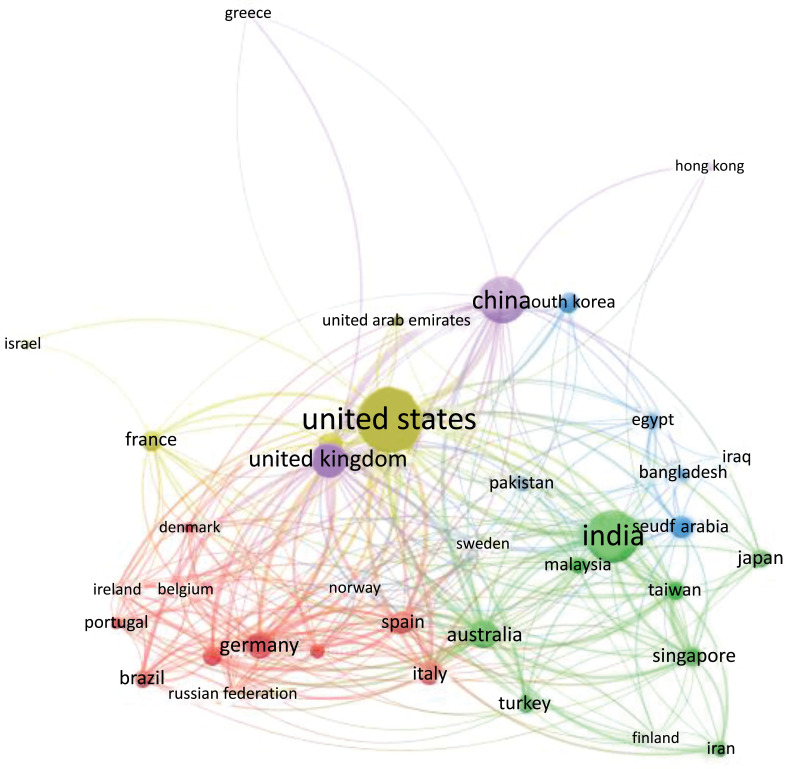
Network of country collaboration.

**Fig. (28):**
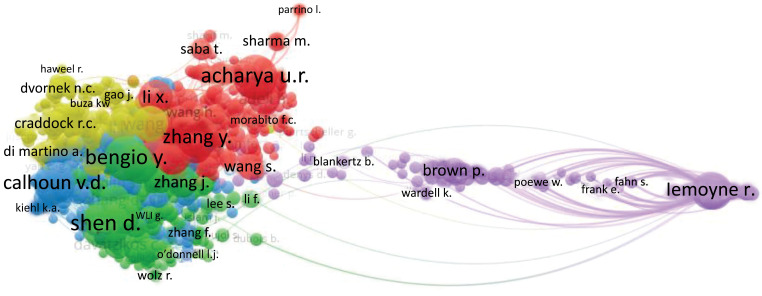
Author co-citation network.

**Table 1:** The sample characteristics.

**Description**	**Result**
**Main Information about the Data**	
Timespan	2015-May 2023
Sources (n)	388
Publications (n)	1550
Average no. of citations per document (n)	15.87
References (n)	403,380
Annual growth rate (%)	36.78
Document Average Age	2.31
**Document Contents**	
Keywords Plus (n)	5010
Author's Keywords (n)	1834
**Authors**	
Authors (n)	3801
Authors of single-authored docs (n)	22
**Authors Collaboration**	
Single-authored docs (n)	44
Co-Authors per Doc (n)	5.67
International co-authorships %	31.43
**Document Types**	
Article (n)	839
Conference paper (n)	389
Review (n)	169
Conference review (n)	64
Book chapter (n)	53
Editorial (n)	20
Book (n)	10
Note (n)	4
Short survey (n)	2

**Table 2:** The most relevant countries.

**Country**	**Articles**	**SCP**	**MCP**	**Freq**	**MCP_Ratio**
USA	128	94	34	0.149	0.266
CHINA	125	71	54	0.145	0.432
INDIA	101	84	17	0.117	0.168
UNITED KINGDOM	29	12	17	0.034	0.586
KOREA	22	18	4	0.026	0.182
AUSTRALIA	21	14	7	0.024	0.333
GERMANY	19	12	7	0.022	0.368
CANADA	17	11	6	0.020	0.353
SPAIN	14	7	7	0.016	0.500

**Note:** *SCP-Single Country Publication, MCP-Multiple Country publication.

**Table 3:** The 10 most globally cited authors.

**Author**	**TC**	**NP**	**PY_start**
CALHOUN VD	858	11	2016
SUI J	674	7	2017
PINAYA WHL	648	7	2016
PLIS S	612	5	2015
MECHELLI A	554	9	2017
LIU M	538	11	2018
ZHANG Y	530	12	2017
ARBABSHIRANI MR	522	1	2017
VIEIRA S	494	7	2017
LI H	470	9	2017

**Table 4:** First 10 author productivity through Lotka’s Law.

**Documents Written**	**No. of Authors**	**Proportion of Authors**
1	3430	0.872
2	344	0.087
3	73	0.019
4	34	0.009
5	18	0.005
6	9	0.002
7	10	0.003
8	4	0.001
9	3	0.001
10	4	0.001

**Table 5:** Top ten the most cited reference on a local level.

**Cited References**	**Citations**
Lecun Y., Bengio Y., Hinton G., Deep Learning, nature, 521, PP. 436-444, (2015) [51]	49
He K., Zhang X., Ren S., Sun J., Deep residual learning for image recognition, proceedings of the IEEE conference on computer vision and pattern recognition, PP. 770-778, (2016) [52]	25
Goodfellow I., Bengio Y., Courville A., Deep learning, (2016) [53]	23
Kingma D.P., Ba J., Adam: A method for stochastic optimization, (2015) [54]	23
Arbabshirani M.R., Plis S., Sui J., Calhoun V.D., Single subject prediction of brain disorders in neuroimaging: Promises and pitfalls, neuroimage, 145, PP. 137-165, (2017) [32]	20
Krizhevsky A., Sutskever I., Hinton G.E., Imagenet classification with deep convolutional neural networks, advances in neural information processing systems, PP. 1097-1105, (2015) [55]	19
Iidaka T., Resting state functional magnetic resonance imaging and neural network classified autism and control, Cortex, 63, PP. 55-67, (2015) [56]	18
Simonyan K., Zisserman A., Very deep convolutional networks for large-scale image recognition, (2015) [57]	17
Heinsfeld A.S., Franco A.R., Craddock R.C., Buchweitz A., Meneguzzi F., Identification of autism spectrum disorder using deep learning and the abide dataset, Neuroimage Clin, 17, PP. 16-23, (2018) [33]	15
Zou L., Zheng J., Miao C., Mckeown M.J., Wang Z.J., 3D CNN based automatic diagnosis of attention deficit hyperactivity disorder using functional and structural MRI, IEEE Access, 5, PP. 23626-23636, (2017) [58]	15

**Table 6:** Future research agenda.

**Area**	**Probable Research Questions**
ML, DL model, and human interaction	How can the relationship between doctors and ML, and DL models be optimized? What is the current level of confidence in ML and DL-based medical models? How can physicians and patients gain faith in ML and DL models? How might ML, DL scientists, and healthcare practitioners best collaborate and collaborate? What role do explainable ML, and DL models play in fostering trust?
Security and robustness	How trustworthy are ML, and DL models trained on other brain disorder datasets? Can adversarial attacks in medicine outwit ML and DL models? How should a brain disorder detection and diagnosis ML, and DL system be developed to be resilient and safe against adversarial assaults and actors? Can a brain disorder detection and diagnosis algorithm be used to identify different forms of brain disorders?
Data storage	To preserve data privacy rights, where should the scan data be stored? Is there a future for emerging technologies like blockchain in the storage and administration of medical data? Should patient data be anonymized permanently or only pseudonymized?
Source of publication collection	Is it worth collecting publications from more than one database like PubMed, Scopus, Web of Science, *etc*.?
Period of publication collection	Is it worth collecting publications over 10 or 15 years for proper bibliometric analysis?
Different language	Is it worth collecting publications in different languages along with English for proper bibliometric analysis?

## LIST OF ABBREVIATIONS

AI: Artificial Intelligence
ASD: Autism Spectrum Disorder
CNN: Convolutional Neural Network
DL: Deep Learning
DNN: Deep Neural Network
FC: Functional Connectivity
H-FCN: Hierarchical Fully Convolutional Network
MCA: Multiple Correspondence Analysis
ML: Machine Learning
MLSP: Machine Learning for Signal Processing
MRI: Magnetic Resonance Imaging
STN: Subthalamic Nucleus
